# Excretory/secretory products from *Trichinella spiralis* adult worms ameliorate myocardial infarction by inducing M2 macrophage polarization in a mouse model

**DOI:** 10.1186/s13071-023-05930-x

**Published:** 2023-10-16

**Authors:** Lingqin Wu, Wenhui Yin, Jutai Wen, Shuying Wang, Huihui Li, Xiaoli Wang, Weixiao Zhang, Shuyao Duan, Qiuyu Zhu, Erhe Gao, Shili Wu, Bin Zhan, Rui Zhou, Xiaodi Yang

**Affiliations:** 1 Anhui Key Laboratory of Infection and Immunity of Bengbu Medical College, Bengbu, 233000 China; 2 First Affiliated Hospital of Bengbu Medical College, Bengbu, 233000 China; 3 Second Affiliated Hospital of Jiaxing University, Jiaxing, 314000 China; 4 Basic Medical College of Bengbu Medical College, Bengbu, 233000 China; 5 Lewis Katz School of Medicine, Temple University, Philadelphia, PA 19140 USA; 6 National School of Tropical Medicine, Baylor College of Medicine, Houston, TX 77030 USA

**Keywords:** Myocardial infarction, Excretory-secretory products, *Trichinella spiralis*, Macrophage, Immunomodulation

## Abstract

**Background:**

Ischemia-induced inflammatory response is the main pathological mechanism of myocardial infarction (MI)-caused heart tissue injury. It has been known that helminths and worm-derived proteins are capable of modulating host immune response to suppress excessive inflammation as a survival strategy. Excretory/secretory products from *Trichinella spiralis* adult worms (*Ts*-AES) have been shown to ameliorate inflammation-related diseases. In this study, *Ts*-AES were used to treat mice with MI to determine its therapeutic effect on reducing MI-induced heart inflammation and the immunological mechanism involved in the treatment.

**Methods:**

The MI model was established by the ligation of the left anterior descending coronary artery, followed by the treatment of *Ts*-AES by intraperitoneal injection. The therapeutic effect of *Ts*-AES on MI was evaluated by measuring the heart/body weight ratio, cardiac systolic and diastolic functions, histopathological change in affected heart tissue and observing the 28-day survival rate. The effect of *Ts*-AES on mouse macrophage polarization was determined by stimulating mouse bone marrow macrophages in vitro with *Ts*-AES, and the macrophage phenotype was determined by flow cytometry. The protective effect of *Ts*-AES-regulated macrophage polarization on hypoxic cardiomyocytes was determined by in vitro co-culturing *Ts*-AES-induced mouse bone marrow macrophages with hypoxic cardiomyocytes and cardiomyocyte apoptosis determined by flow cytometry.

**Results:**

We observed that treatment with *Ts*-AES significantly improved cardiac function and ventricular remodeling, reduced pathological damage and mortality in mice with MI, associated with decreased pro-inflammatory cytokine levels, increased regulatory cytokine expression and promoted macrophage polarization from M1 to M2 type in MI mice. *Ts*-AES-induced M2 macrophage polarization also reduced apoptosis of hypoxic cardiomyocytes in vitro.

**Conclusions:**

Our results demonstrate that *Ts*-AES ameliorates MI in mice by promoting the polarization of macrophages toward the M2 type. *Ts*-AES is a potential pharmaceutical agent for the treatment of MI and other inflammation-related diseases.

**Graphical Abstract:**

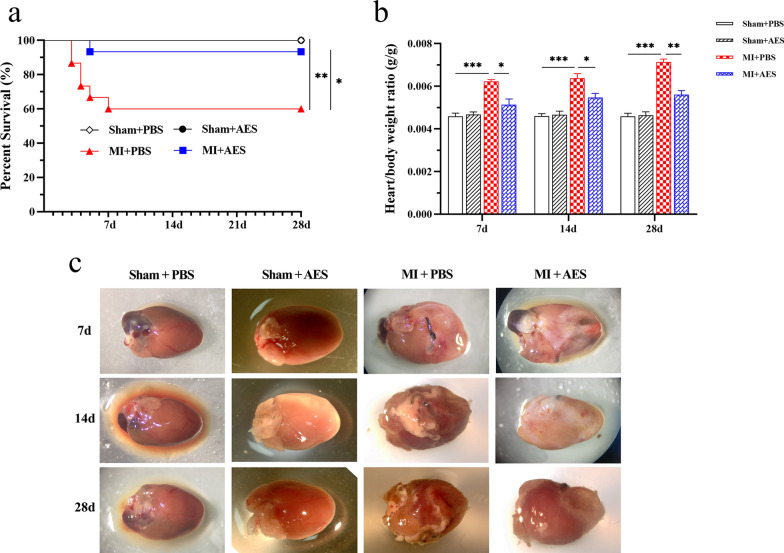

## Background

Myocardial infarction (MI) is a heart attack caused by the interruption or blockage of blood flow into heart tissue, leading to myocardial cell necrosis and heart failure, with high morbidity and mortality worldwide [1, 2]. It even becomes a common cause of premature death in young and middle-aged populations [3, 4]. The mortality associated with MI was also high during the COVID-19 pandemic [5, 6].

If survived from the sudden death of heart failure, the pathological process of MI includes three consecutive phases: inflammation, proliferation and maturation [7–9]. The inflammatory phase is characterized by the rapid sterile inflammation [7]. After myocardial hypoxia injury, necrotic cells release inflammatory signals that activate immune pathways such as the stimulation of Toll-like receptor signaling pathway and complement activation, which in return trigger an intense inflammatory response. Similar to microbially induced inflammation, MI caused sterile inflammation also recruits neutrophils and macrophages that produce pro-inflammatory cytokines and chemokines, notably tumor necrosis factor (TNF) and interleukin-1 (IL-1) [10]. Immune cell infiltration and phagocytosis help remove damaged cells and extracellular matrix [11–13], followed by a proliferative phase that includes the transformation of the pro-inflammatory response into anti-inflammatory response and fibroblast activation into myofibroblasts, scar formation and angiogenesis [14]. Avoiding the overreaction of the inflammation and timely transition of the inflammatory phase to the proliferative and mature phases is critical for the prognosis of MI. Excessive infiltration of inflammatory cells and release of pro-inflammatory cytokines or reactive oxygen species (ROS) into the injured heart tissue could exacerbate myocardial injury and worsen the myocardial remodeling process [7, 15].

Macrophage is one of the most abundant cell types infiltrated in the infarcted heart [16] and plays an important role in the initiating inflammatory responses and the afterward healing process of MI. Upon occurrence of MI, many blood monocytes infiltrate into the ischemic heart tissue and differentiate into macrophages [17]. Activated macrophages can be classified into two main categories: classically activated macrophage (M1 type) and alternative activated type (M2) [18, 19]. In the early pathogenesis of MI, M1 macrophages produce several pro-inflammatory cytokines, such as IL-1β, IL-6, IL-12, TNF-α and chemokines involved in immune cell recruitment and inflammation responses [11, 20]. In the middle and late stages of MI, M1 macrophages are transformed to M2 macrophages leading to the subsidence of inflammation and the initiation of reparative/proliferative phase. M2 macrophages secrete regulatory cytokines such as TGF-β and IL-10, chemokines (CCL17, CCL22, CCL2) and arginase-1 (Arg-1), which reduce inflammation and stimulate cell proliferation, angiogenesis, extracellular matrix formation and collagen synthesis, which facilitate tissue repair [16, 21, 22]. Therefore, induction of macrophage polarization from M1 to M2 phenotype and modulation of M1 and M2 balance is a feasible therapeutic approach to relieve MI and reduce MI mortality.

In recent years, “worm therapy” has attracted much attention. Worms and their derived proteins have been shown therapeutic effects on a variety of inflammatory or autoimmune diseases such as diabetes [23], multiple sclerosis [24], allergic rhinitis [25], systemic lupus erythematosus [26] and encephalomyelitis [27]. These effects are mainly achieved through immunomodulating host immune responses to inhibit inflammatory responses and promote regulatory immune responses including induction of M2 macrophages [28, 29]. The helminth infection or derived products stimulate M2 macrophage polarization, which inhibits excessive inflammatory responses and promotes the healing and repair of the tissue damage caused by microbial infections [30, 31] or metabolic diseases [32]. *Schistosoma japonicum* secreted cysteine protease inhibitor (*Sj*-Cys) effectively alleviated sepsis-induced myocardial injury, indicating that helminth-derived proteins could protect myocardial injury caused by the excessive inflammation [33].

*Trichinella spiralis* is an intestinal nematode with larvae dwelling in transverse muscle. To facilitate their survival in the hostile environment of the host, the adult worms or muscle larvae secrete a variety of proteins to immunomodulate host immune responses [34]. In our previous studies, we have demonstrated that extracts from *T. spiralis* adult worms reduced allergic inflammation in an experimental asthma mouse model [35]. The ES products derived from adult worms ameliorated DSS-induced colitis [36]. Interestingly, both ES products derived from *T. spiralis* adult worms or muscle larvae could protect mice from sepsis-induced heart injury [37] and lung injury [31]. In this study, we aimed to determine whether *T. spiralis* adult ES products (*Ts*-AES) possess the ability to reduce MI-induced sterile myocardial inflammation/damage and understand the immunological mechanism associated with the therapeutic effect.

## Methods

### Animals

The specific pathogen-free male C57BL/6 J mice (8–10 weeks old with weight of 18–22 g), female ICR mice (6–8 weeks old with weight of 25–30 g) and female Wistar rats (6 weeks old with weight of 180–220 g) were purchased from the Experimental Animal Center of Bengbu Medical College and maintained in a controlled environment (12:12 h light/dark photocycle with temperature of 22 ± 2 °C and relative humidity of 55%). All animals received a normal diet and free access to water. The animal experimental procedures comply with the animal ethics standards of Bengbu Medical College and are approved by the ethics committee.

### Preparation of *Ts*-AES

*Trichinella spiralis* muscle larvae were isolated from the muscles of female ICR mice infected with 800 larvae for 45 days using the method of modified pepsin-hydrochloric acid digestion [38]. The collected muscle larvae were used to orally infect Wistar rats (12,000–15,000 larvae per rat). The adult worms were collected from the intestines 84 h after infection and cultured in RPMI-1640 medium supplemented with 100 U/ml penicillin and 100 µg/ml streptomycin at 37 °C, 5% CO_2_ for 48 h. The culture supernatant containing *Ts*-AES was collected and concentrated by centrifugating with an ultrafiltration tube and buffer exchanged into PBS. The potential contaminated endotoxin in *Ts*-AES products was removed using a ToxOut™ High Capacity Endotoxin Removal Kit (BioVision, Palo Alto, CA, USA), and low endotoxin level was confirmed by using ToxinSensor™ Chromogenic Limulus Amebocyte Lysate (LAL) Endotoxin Assay Kit (GenScript Biotechnology, Nanjing, China) following the manufacturer’s protocol. The protein concentration of the collected *Ts*-AES was determined using Bicinchoninic Acid Protein Assay Kit (Beyotime, China) and the prepared *Ts*-AES stored at -80 °C until use.

### Development of a myocardial infarction mouse model

The MI mouse model was established by the ligation of the left anterior descending (LAD) coronary artery as described [39]. Mice were anesthetized under isoflurane inhalation. An incision of approximately 5 mm length was made on the left side of the chest between the 4th–5th rib of the mice. After opening the pericardium, the LAD was located and ligated 3 mm from its origin using a 6–0 silk suture. The successful ligation was confirmed by seeing the anterior wall of the left ventricle turn pale. The mice in sham group underwent a similar surgical procedure without LAD ligated.

### Treatment of myocardial infarction with *Ts*-AES

Total 132 C57BL/6 J mice were randomly divided into four groups (33/group): (i) MI mice treated with *Ts*-AES (MI + AES); (ii) MI mice treated with PBS (MI + PBS); (iii) sham-operated mice treated with AES (Sham + AES); (iv) sham-operated mice treated with PBS (Sham + PBS). The mice were each treated intraperitoneally with 25 µg *Ts*-AES in a total volume of 100 µl or the same volume of PBS 30 min after surgery and continued the treatment on day 2, 4 and 6 post surgery. All mice in each group underwent echocardiography to examine cardiac function on postoperative day 7, 14 and 28. Six mice from each group were killed after each echocardiogram, and the ocular blood and hearts were collected. The sera were separated and stored at − 20 °C. Each heart was weighed, and the heart/body weight ratio was calculated. The survival rate of the remaining mice in each group (15 mice) was observed for 28 days.

### Cytokine measurement

The levels of IL-6, TNF-α, TGF-β and IL-10 in sera of mice were measured using the LEGEND MAX™ ELISA kit (Dakewe Biotech, China).

### Echocardiography

The echocardiography was performed on mice using an animal visual ultrasound imaging system with mouse probes (VisualSonics, Canada, and MS-400 probe) as previously described [40]. Each mouse was fixed on a thermostat in supine position with electrodes attached on limbs. Superficial anesthesia was maintained with 1% isoflurane and oxygen. Long-axis B Mode was used to acquire images of the left ventricle and M-motion curves of the left ventricular wall were collected for at least three continuous and stable cardiac cycles. Left ventricular trace method was used to reveal the following parameters: stroke volume (SV), left ventricular ejection fraction (LVEF), left ventricular fractional shortening (LVFS), left ventricular end systolic volume (LVESV) and left ventricular end diastolic volume (LVEDV). All data were averaged from three cardiac cycles. The positions of mitral valve orifice and ventricular septal were judged by M-mode ultrasound, the flow spectrum of mitral valve orifice was observed by pulse spectrum Doppler, and the myocardial motion spectrogram of the mitral annulus of the interventricular septum was collected by tissue Doppler module. The following parameters such as the peak value of early diastolic blood flow (E peak), the peak value of late diastolic blood flow (A peak), isovolumic contraction time (IVCT), isovolumic relaxation time (IVRT) and ejection time (ET) were measured to obtain E/A values and left ventricular myocardial performance index (LV MPI) in three cardiac cycles [41]. All operations were performed by the same person, and the mouse heart rate was maintained consistent when images were acquired in consent mode to exclude manipulation errors and the influence of heart rate on experimental results.

### Hematoxylin and eosin (H&E) staining and Masson’s trichrome staining of heart tissue

The hearts collected from each group of mice at different time points (7, 14, 28 days post surgery) were fixed in 4% paraformaldehyde and embedded in paraffin blocks. The myocardial tissue was sliced to 5-μm-thick sections. H&E staining (Servicebio, China) or Masson’s trichrome staining (Servicebio, China) was performed on sections as described [42]. The histopathological changes and level of fibrosis were observed under a microscope (Nikon, Japan).

### RNA extraction and real-time quantitative PCR (RT-qPCR)

Total RNA was extracted from cardiac infarction zones or the same location region in groups of Sham + AES and Sham + PBS by using TRIzol reagent (TransGen Biotech, China) and quantified by OD_260_. The total cDNAs were reversely transcribed from 2 µg total RNA using the Revert Aid First Strand cDNA Synthesis Kit (TransGen Biotech, China). The relative mRNA expression of different cytokines (IL-6, TNF-α, IL-10 and TGF-β), macrophage polarization-related markers (iNOS and CD206), myofibroblast markers (α-SMA) and vascular endothelial growth factor (VEGF) in heart tissue was determined using PerfectStart®Green qPCR SuperMix (TransGen Biotech, China) on Roche LightCycler® 96 real-time PCR system (Roche Molecular Systems, USA) using the corresponding primers listed in Table 1. The relative mRNA expression was calculated by the formula 2^−△△Ct^ compared with housekeeping GAPGH.

**Table 1:** Related primers of target genes used in qPCR

ID	Primer sequences (5’ → 3’)
GAPDH-F	GGTTGTCTCCTGCGACTTCA
GAPDH-R	TGGTCCAGGGTTTCTTACTCC
IL-6-F	GTCCTTCCTACCCCAATTTCCA
IL-6-R	TAACGCACTAGGTTTGCCGA
TNF-α-F	CGAGTGACAAGCCTGTAGCC
TNF-α-R	ACAAGGTACAACCCATCGGC
IL-10-F	GGTTGCCAAGCCTTATCGGA
IL-10-R	AATCGATGACAGCGCCTCAG
TGF-β-F	CTGGATACCAACTACTGCTTCAG
TGF-β-R	TTGGTTGTAGAGGGCAAGGACCT
iNOS-F	CAAGCACCTTGGAAGAGGAG
iNOS-R	AAGGCCAAACACAGCATACC
CD206-F	AAGGTAAGCAAGTCTCCCATTC
CD206-R	TGACACCCAGCGGAATTTC
ɑ-SMA-F	AGCGGGCATCCACGAAAC
ɑ-SMA-R	TTGATCTTCATGGTGCTGGGT
VEGF-F	CCCACGTCAGAGAGCAACAT
VEGF-R	TGCGCTTTCGTTTTTGACCC

### Induction of mouse bone marrow-derived macrophages (BMDMs)

The femoral and tibial bones were separated from killed C57BL/6J mice, and the bone marrow cells were extracted by flushing the bone marrow cavities with complete DMEM medium (Gibco, USA) containing 10% fetal bovine serum (FBS) (Every Green, China) and penicillin (100 U/ml)/streptomycin (100 µg/ml) (Beyotime Biotechnology, China). The cell suspension was filtered through a 200-mesh screen to remove debris and washed twice. The collected bone marrow cells were cultured in a complete DMEM medium containing 20 ng/ml murine macrophage colony-stimulating factor (M-CSF) (R&D Systems, USA) for 7 days. The cells adhered on the plates were collected as matured BMDMs.

### Effect of* Ts*-AES on macrophage polarization in vitro

The matured BMDMs obtained above were divided into four groups with 1 × 10^6^ cells for each group: (i) BMDMs incubated with *Ts*-AES (4 μg/ml) (AES + Mφ); (ii) BMDMs incubated with LPS (100 ng/ml) (Solarbio, China) and IFN-γ (10 ng/ml) (R&D Systems, USA) (LPS + IFN-γ + Mφ); (iii) BMDMs incubated with LPS (100 ng/ml) and IFN-γ (10 ng/ml) in the presence of *Ts*-AES (4 μg/ml) (AES + LPS + IFN-γ + Mφ); (iv) BMDMs incubated with PBS as the control group (PBS + Mφ). After being incubated for 24 h, cells were collected, and M1 macrophage-associated marker CD86 and M2 macrophage-associated marker CD206 expressed on the surface of the cells were measured by flow cytometry.

### Flow cytometry

To differentiate the live from the dead cells, cultured BMDM cells were treated with fixable viability dye efluor 510 (BioLegend, USA) for 10 min. After being blocked with Fc receptor blocker (BioLegend, USA) for 10 min, the cells were incubated with FITC-anti-F4/80 (BioLegend, USA), BV605-anti-CD11b (BioLegend, USA) and APC-anti-CD86 (Thermo Fisher Scientific, USA) antibodies for 25 min. To stain CD206, the cells were fixed and permeabilized using a Thermo Fixation/Permeabilization Kit (Thermo Fisher Scientific, USA) and stained with PE-anti-CD206 (BioLegend, USA) for 30 min.

The ratio of apoptotic hypoxic cardiomyocyte cells was determined using Annexin V-AbFluor™/PI (Bestbio, China). Cells were harvested and suspended in binding buffer and then stained with Annexin V for 15 min and PI for 5 min at room temperature in dark conditions. The flow cytometry for these stained cells was performed by a DxP Athena™ flow cytometer (Cytek Biosciences, USA). Data were calculated using FlowJo software v10.5 (FlowJo LLC).

### Culture of primary cardiomyocytes

The primary mouse cardiomyocytes were obtained as described [43]. Briefly, the hearts were aseptically collected from killed infant C57BL/6 mice (1–3 days old), chopped into pieces and then digested in D-HankS solution (Gibco, USA) containing trypsin (Gibco, USA), collagenase (Solarbio, China) and DNase I (Solarbio, China) [43] at 37 °C for 10 min. The digestion was performed five times until single heart cells were obtained. The single heart cells were cultured in DMEM-F12 (Gibco, USA) containing 10% FBS (Every Green, China) at 37 ℃, 5% CO_2_, for 2 h. The adherent cells (mostly the fibroblasts) were removed, and the suspended cells were cultured in 4 ml DMEM-F12 complete medium containing 0.1 mmol/l 5-bromo-2-deoxyuridine (Sigma-Aldrich, Germany) for 48 h to inhibit fibroblast growth. The cells were cultured in complete medium without 5-bromo-2-deoxyuridine for another 24–48 h to obtain primary cardiomyocytes.

### Effects of *Ts*-AES-regulated macrophage polarization on hypoxic cardiomyocytes in vitro

Primary cardiomyocytes obtained above were seeded in six-well plates with 1 × 10^6^/well in low-glucose DMEM medium without FBS. The mature bone marrow-derived macrophage cells (BMDMs) were pre-treated in medium with *Ts*-AES (4 μg/ml) or LPS (100 ng/ml) + IFN-γ (10 ng/ml), washed and seeded on the bottom surface of the Transwell chambers with pore size of 0.4 μm (Corning, USA) at 2 × 10^5^/well and then inserted into the six-well plate seeded with primary cardiomyocytes [44]. The culture environment was adjusted to anaerobic condition in a culture bag filled with anaerobic gas (Mitsubishi Gas Chemical, Japan) with an oxygen content indicator strip (Mitsubishi Gas Chemical, Japan) inside [45]. The oxygen content in the bag should be < 1%. After being incubated for 24 h at anaerobic condition, cells were collected to measure cardiomyocyte apoptosis using flow cytometry; the culture supernatants were collected to measure the concentration of IL-6, TNF-α, TGF-β and IL-10 using LEGEND MAX™ELISA kits (Dakewe Biotech, China).

### Statistical analysis

GraphPad Prism version 7 software (GraphPad Software, Inc., USA) was used to analyze statistical differences between groups. Results are presented as means ± SEM. Statistical analysis was performed using the Shapiro-Wilk normality test and one-way analysis of variance (ANOVA) followed by Tukey-Kramer multiple comparisons test or unpaired two-tailed Student’s t-test. The difference in survival rates among the groups was compared using Chi-square test. *P* < 0.05 was considered statistically significant.

## Results

### *Ts*-AES alleviated myocardial infarction in mice

#### *Ts*-AES improved the survival rate of mice with myocardial infarction

**Fig.1:**
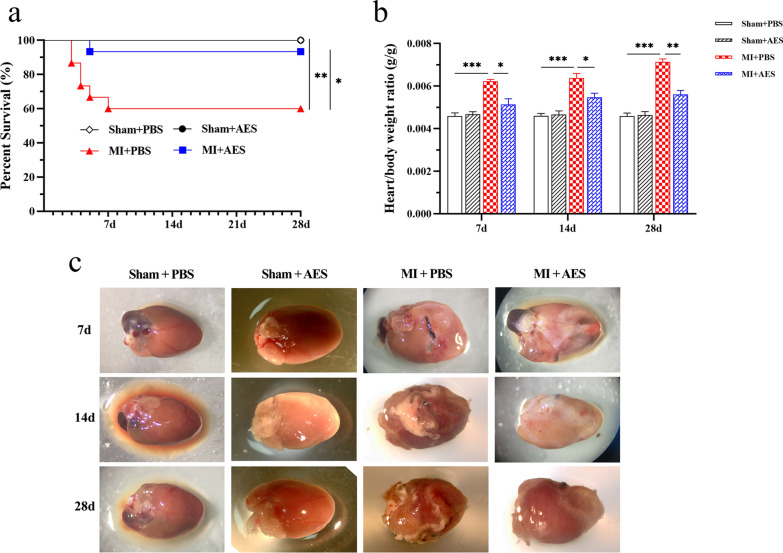
*Ts*-AES significantly mitigates MI by improving the survival rate up to 28 days, reducing the ratio of heart to body weight. **a** *Ts*-AES significantly increased the survival rate of mice with MI up to 28 days (93.3%) compared to mice receiving PBS (60%) during the same observation period (*n* = 15). **b** *Ts*-AES reduced the heart-to-body weight ratio of mice with MI (*n* = 6). **c** The representative heart pictures from different treatment groups under asana microscopy. The results are presented as mean ± SEM, *P < 0.05, **P < 0.01, ***P < 0.001

The MI mouse model was established by ligating left anterior descending (LAD) coronary artery. To evaluate the therapeutic effect of *Ts*-AES on mice with MI, the mice with MI surgery were treated intraperitoneally with 25 µg *Ts*-AES for each mouse. The 28-day survival rate of *Ts*-AES-treated group (MI + AES) reached 93.3% (14/15), which is significantly higher than the 60% (9/15) survival rate of MI group without treatment (MI + PBS) (Chi-square test, *χ*^*2*^ = 4.714, *df* = 1, *P* = 0.0299) (Fig. 1a). Most deaths happened within the first week post MI surgery. All mice in sham-operated mice treated with either *Ts*-AES (Sham + AES) or PBS (Sham + PBS) survived up to 28 days. The ratio of heart weight to body weight was also significantly reduced in the MI group treated with *Ts*-AES compared with those without treatment at 7, 14 and 28 days post MI surgery, with the most significant difference at 28 days (7d: ANOVA, *F*_(3, 20)_ = 19.26, *P* < 0.0001; 14d: ANOVA, *F*_(3, 20)_ = 22.07, *P* < 0.0001; 28d: ANOVA, *F*_(3, 20)_ = 54.73, *P* < 0.0001) (Fig. 1b). The gross pathology observation on hearts collected 28 days post treatment revealed that the infarct zone was clearly visible in hearts from untreated MI mice (MI + PBS), while the infarct zone was lighter and smaller in mice treated with *Ts*-AES (MI + AES) (Fig. 1c). These results suggest that treatment with *Ts*-AES significantly mitigates MI by improving the survival rate up to 28 days, reducing the gross pathology in affected hearts.

#### *Ts*-AES improved cardiac function in mice with MI

**Fig. 2:**
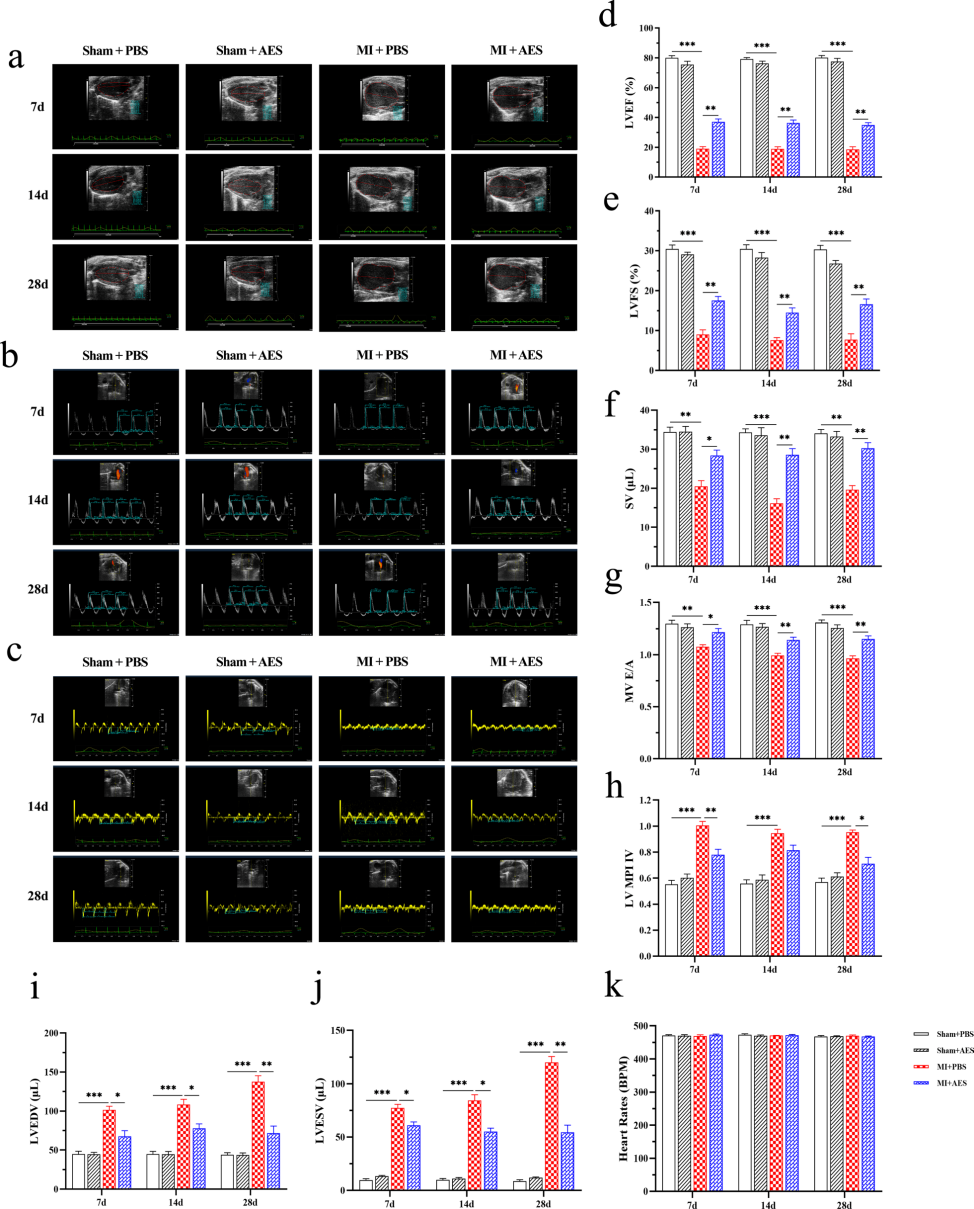
Cardiac functional changes in different groups of mice measured by cardiac echocardiogram at 7d, 14d and 28d post MI surgery, showing *Ts*-AES significantly improved systolic and diastolic heart function in mice with MI. **a**–**c** Representative echocardiographic changes in long axis B mode, pulse spectrum Doppler and tissue Doppler in each group of mice, respectively. **d**–**J** Changes in systolic and diastolic functions (LVEF, LVFS, SV, MV E/A; LV MPI IV, LVEDV, LVESV, respectively) in mice with MI. **k** Heart rate of mice in each group when images were acquired. The results were presented as mean ± SEM (*n* = 6), *P < 0.05, **P < 0.01, ***P < 0.001

Echocardiograms were performed in each mouse from each group on the 7th, 14th and 28th postoperative days. The echocardiogram in the long-axis B mode showed that untreated MI mice (MI + PBS) revealed significantly larger heart chambers that are pear or spherical shaped, and the anterior and posterior wall motion of the left ventricle was significantly reduced, whereas the MI mice treated with *Ts*-AES (MI + AES) had smaller heart chambers and significantly improved wall motion (Fig. 2a). Pulse spectrum Doppler (Fig. 2b) and tissue Doppler (Fig. 2c) also showed that E peak was inverted to A peak in mice with MI on the 14th and 28th day post-surgery. Treatment with *Ts*-AES reduced the E/A peak inversion. The impaired left ventricular systolic and diastolic functions were also observed in mice with MI showing decreased LVEF, LVFS, SV and E/A and increased LVEDV, LVESV and LV MPI (7d: ANOVA, *F*_(3, 20)_ = 277.1, *P* < 0.0001; *F*_(3, 20)_ = 32.91, *P* < 0.0001; *F*_(3, 20)_ = 24.28, *P* < 0.0001; *F*_(3, 20)_ = 10.51, *P* = 0.0002; *F*_(3, 20)_ = 30.04, *P* < 0.0001; *F*_(3, 20)_ = 183.6, *P* < 0.0001; *F*_(3, 20)_ = 37.03, *P* < 0.0001; 14d: ANOVA, *F*_(3, 20)_ = 379.9, *P* < 0.0001; *F*_(3, 20)_ = 101.6, *P* < 0.0001; *F*_(3, 20)_ = 32.75, *P* < 0.0001; *F*_(3, 20)_ = 21.87, *P* < 0.0001; *F*_(3, 20)_ = 36.37, *P* < 0.0001; *F*_(3, 20)_ = 124.2, *P* < 0.0001; *F*_(3, 20)_ = 29.95, *P* < 0.0001; 28d: ANOVA, *F*_(3, 20)_ = 331.3, *P* < 0.0001; *F*_(3, 20)_ = 74.75, *P* < 0.0001; *F*_(3, 20)_ = 29.93, *P* < 0.0001; *F*_(3, 20)_ = 30.79, *P* < 0.0001; *F*_(3, 20)_ = 48.93, *P* < 0.0001; *F*_(3, 20)_ = 137.4, *P* < 0.0001; *F*_(3, 20)_ = 25.35, *P* < 0.0001, respectively). Treatment with *Ts*-AES significantly improved LV functions observed at postoperative day 7, 14 and 28 measured by these parameters (Fig. 2d–j). No systolic or diastolic functional change was observed in mice with sham operation treated with either *Ts*-AES (Sham + AES) or PBS (Sham + PBS). The heart rate of the mice was maintained consistent at the time of echocardiographic acquisition in consent mode, with no significant difference between groups to ensure that the heart function of the mice was not affected by change of heart rate (7d: ANOVA, *F*_(3, 20)_ = 0.2427, *P* = 0.8655; 14d: ANOVA, *F*_(3, 20)_ = 0.4449, *P* = 0.7235; 28d: ANOVA, *F*_(3, 20)_ = 0.1775, *P* = 0.9104) (Fig. 2k). All results suggest that *Ts*-AES significantly improves the cardiac dysfunction of mice with MI.

### *Ts*-AES reduced myocardial damage and inflammatory cell infiltration in heart with MI

**Fig. 3:**
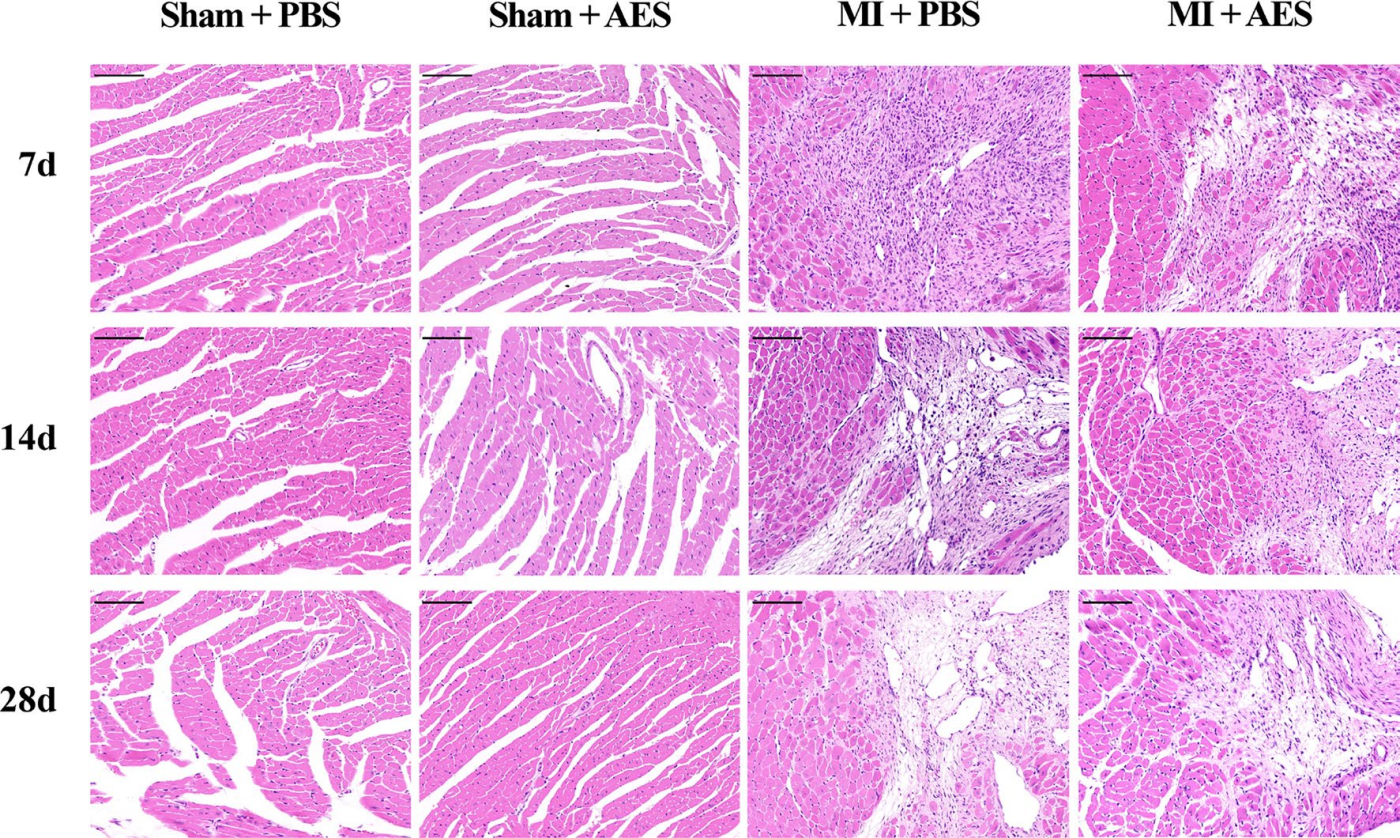
*Ts*-AES alleviated myocardial damage in mice with MI. Representative sections of H&E staining of the MI junction area in each group (200 ×, scale bar: 100 μm)

The degree of myocardial damage and inflammatory cell infiltration in the interface area of MI in mice was observed by histochemical examination with H&E staining. The results showed that many inflammatory cells were significantly infiltrated in the interface area of MI in mice with MI surgery (MI + PBS). Many myocardial cells were vacuolated, and the myocardial fibers were broken. In mice treated with *Ts*-AES (MI + AES), the infiltration of inflammatory cells and damage of myocardial cells were significantly reduced compared with the group without treatment. No pathological change was seen in Sham surgery groups with or without treatment of *Ts*-AES (Fig. 3). The histopathological results demonstrated that *Ts*-AES was able to alleviate MI caused myocardial damage by decreasing inflammatory cell infiltration in MI mice and reducing dead myocardial cells.

### *Ts*-AES inhibited excessive myocardial fibrosis and promoted ventricular remodeling in MI mice

**Fig. 4:**
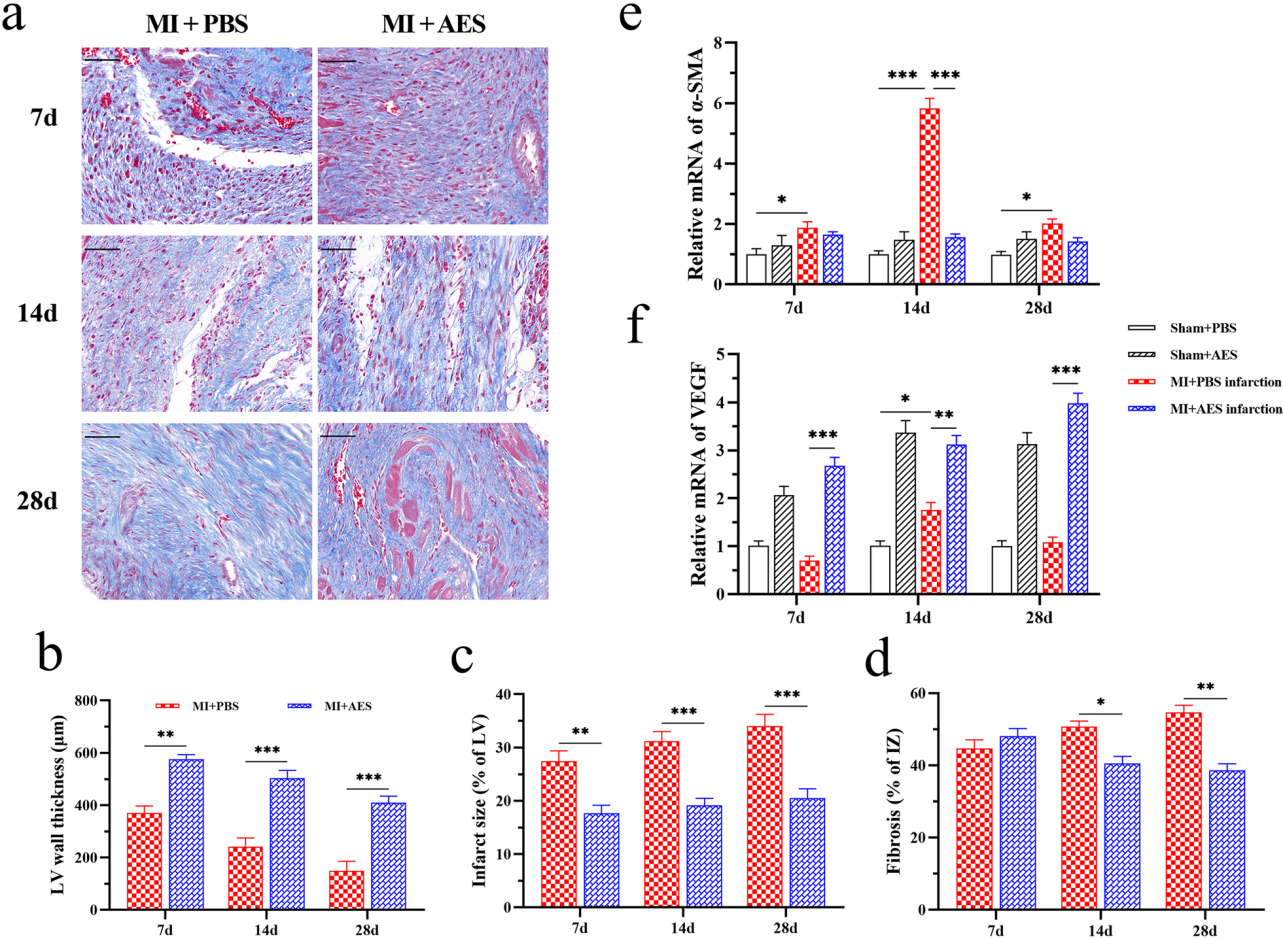
*Ts*-AES inhibited excessive myocardial fibrosis and promoted ventricular remodeling after MI in mice. **a** Representative sections of Masson staining of infarcted heart tissue in each group after MI (400 × , Scale bar: 50 μm). **b** Thickness of the left ventricular wall in each group (*n* = 4). **c** Percentage infarction area of LV in each group (*n* = 4). **d** Fibrosis area of the infarction zone (IZ) in each group (*n* = 4). **e** mRNA transcriptional levels of α-SMA in the infarction zone (*n* = 6). **f** mRNA transcriptional levels of VEGF in infarct region of heart in each treated group (*n* = 6). The results are presented as mean ± SEM, *P < 0.05, **P < 0.01, ***P < 0.001

The histopathological examination of affected heart tissue revealed the therapeutic effect of *Ts*-AES on the healing of MI caused heart damage characterized by the reduced fibrosis stained with Masson and reduced disrupted heart tissue (Fig. 4a), significantly increased wall thickness of the LV infarct area (7d: *t*-test, *t*_(6)_ = 6.463, *P* = 0.0007; 14d: *t*-test, *t*_(6)_ = 5.792, *P* = 0.0012; 28d: t-test, *t*_(6)_ = 6.086, *P* = 0.0009) (Fig. 4b) and reduced LV infarct area at the 7th, 14th and 28th days after MI surgery (7d: t-test, *t*_(6)_ = 3.957, *P* = 0.0075; 14d: *t*-test, *t*_(6)_ = 5.266, *P* = 0.0019; 28d: *t*-test, *t*_(6)_ = 4.870, *P* = 0.0028) (Fig. 4c) compared to the group without treatment of *Ts*-AES (MI + PBS). The Masson staining revealed that there was no visible change in the size of fibrosis at day 7, but it was significantly decreased in the *Ts*-AES treated group at days 14 and 28 compared to the PBS control (7d: *t*-test, *t*_(6)_ = 1.045, *P* = 0.3364; 14d: *t*-test, *t*_(6)_ = 4.209, *P* = 0.0056; 28d: *t*-test, *t*_(6)_ = 5.814, *P* = 0.0011) (Fig. 4d). These results suggested that *Ts*-AES reduced excessive fibrosis and facilitated ventricular remodeling after suffering MI.

After MI, myofibroblast-secreted α-SMA is involved in the fibrosis of infarcted heart tissue and VEGF involved in revascularization to facilitate the repair of infarcted myocardium. To investigate whether *Ts*-AES alleviates myocardial hyperfibrosis and promotes revascularization after MI, the mRNA expression of α-SMA and VEGF in the infarcted area of MI mice was detected by RT-qPCR. The results showed that α-SMA transcriptional expression in the infarcted heart tissue was elevated after MI surgery and reached to the highest at day 14. However, after being treated with *Ts*-AES, the α-SMA transcriptional expression level was significantly decreased at day 14 after MI surgery (MI + AES) compared to group without treatment (MI + PBS) (7d: ANOVA, *F*_(3, 20)_ = 3.147, *P* = 0.0478; 14d: ANOVA, *F*_(3, 20)_ = 101.0, *P* < 0.0001; 28d: ANOVA, *F*_(3, 20)_ = 6.653, *P* = 0.0027) (Fig. 4e). Contrarily, the transcriptional expression of VEGF was significantly increased soon after being treated with *Ts*-AES and maintained at the high level up to 28 days after MI, even in both MI or sham surgery groups with *Ts*-AES treatment (7d: ANOVA, *F*_(3, 20)_ = 40.07, *P* < 0.0001; 14d: ANOVA, *F*_(3, 20)_ = 38.10, *P* < 0.0001; 28d: ANOVA, *F*_(3, 20)_ = 73.54, *P* < 0.0001) (Fig. 4f). The results suggest that *Ts*-AES may contribute to the reduced myocardial fibrosis and increased revascularization in infarcted heart tissue.

### *Ts*-AES reduced inflammatory response in infarcted heart tissue by inducing M2 macrophage polarization

**Fig. 5:**
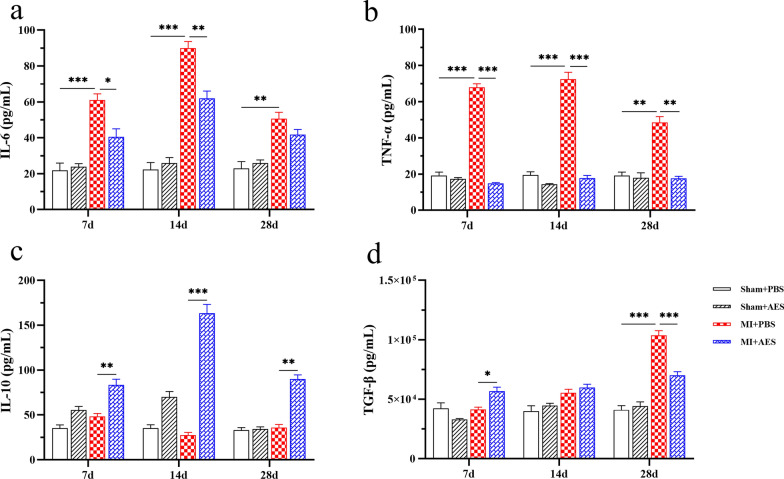
*Ts*-AES systemically reduced pro-inflammatory cytokine levels (IL-6, TNF-α) and increased IL-10 and TGF-β in sera of mice with MI (*n* = 6). The results are presented as mean ± SEM, *P < 0.05, **P < 0.01, ***P < 0.001

To determine whether inflammatory cytokines are involved in the developmental course and prognosis of MI, the levels of inflammatory cytokines IL-6 and TNF-α were measured in sera of mice with MI surgery. The results revealed serological levels of IL-6 and TNF-α were significantly elevated in mice 7 days after MI surgery, and the high levels remained up to 28 days after MI occurred. However, these pro-inflammatory cytokines were significantly decreased after being treated with *Ts*-AES compared to those without treatment (7d: ANOVA, *F*_(3, 20)_ = 25.79, *P* < 0.0001; *F*_(3, 20)_ = 329.5, *P* < 0.0001; 14d: ANOVA, *F*_(3, 20)_ = 74.64, *P* < 0.0001; *F*_(3, 20)_ = 154.8, *P* < 0.0001; 28d: ANOVA, *F*_(3, 20)_ = 17.58, *P* < 0.0001; *F*_(3, 20)_ = 40.57, *P* < 0.0001, respectively) (Fig. 5a, b). There was no change for these inflammatory cytokines in mice with sham surgery with or without *Ts*-AES. The results indicate that *Ts*-AES specifically reduces MI-induced inflammation. On the other hand, treatment with *Ts*-AES significantly increased the levels of IL-10 in sera of mice with MI surgery, reaching the peak 14 days after surgery (7d: ANOVA, *F*_(3, 20)_ = 20.21, *P* < 0.0001; 14d: ANOVA, *F*_(3, 20)_ = 101.2, *P* < 0.0001; 28d: ANOVA, *F*_(3, 20)_ = 65.12, *P* < 0.0001) (Fig. 5c). The level of TGF-β was also increased in the sera of mice at the early stage of MI (7 days post MI surgery), but no increase or even decrease was observed in the late stage of MI (7d: ANOVA, *F*_(3, 20)_ = 10.63, *P* = 0.0002; 14d: ANOVA, *F*_(3, 20)_ = 7.930, *P* = 0.0011; 28d: ANOVA, *F*_(3, 20)_ = 66.11, *P* < 0.0001) (Fig. 5d).

**Fig. 6:**
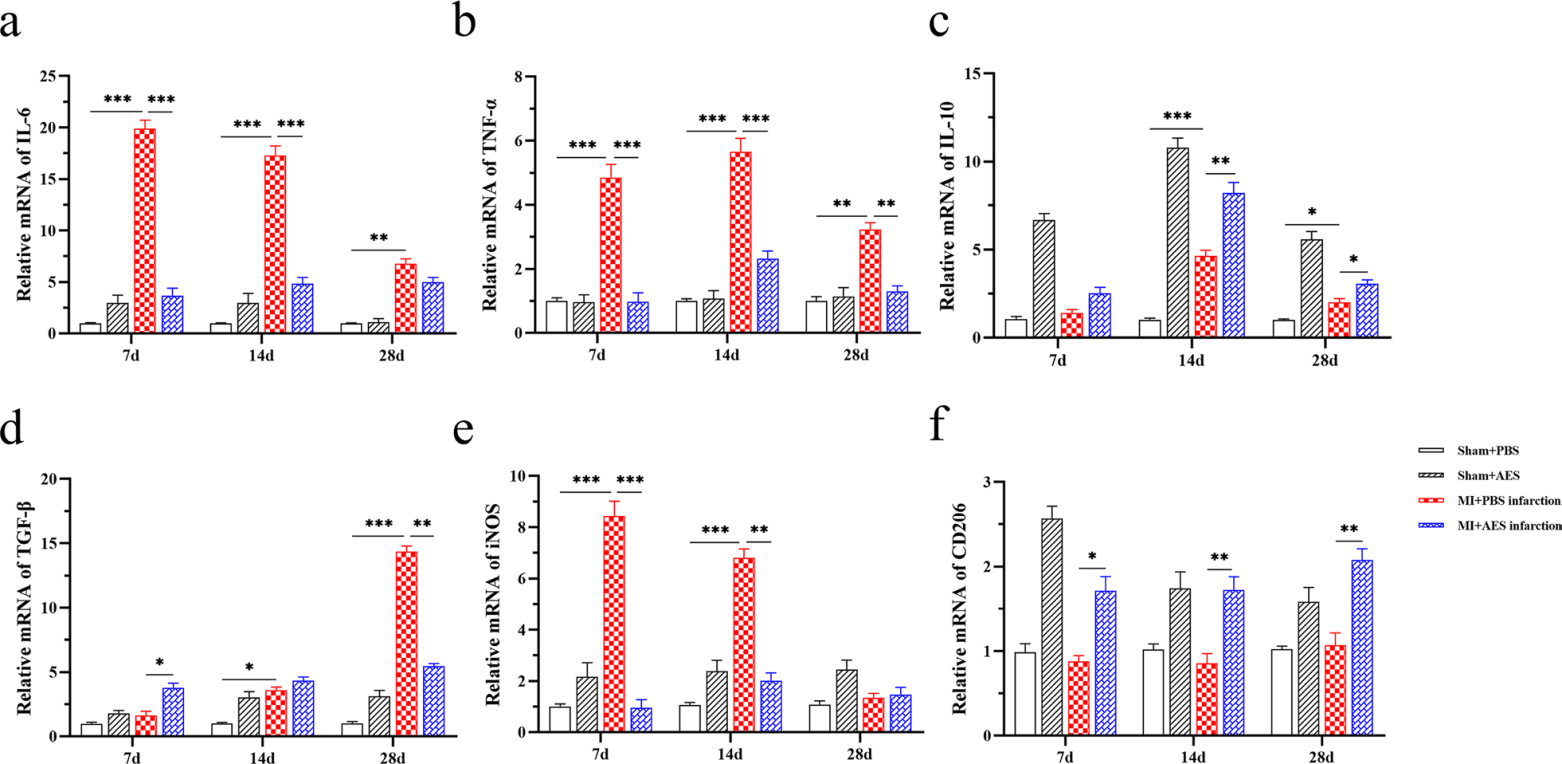
*Ts*-AES regulated the mRNA expression of pro-inflammatory or anti-inflammatory cytokines in the infarct region of mice with MI and promoted the polarization of macrophages to M2 type (*n* = 6). The results are presented as mean ± SEM, *P < 0.05, **P < 0.01, ***P < 0.001

Similar effects of *Ts*-AES treatment on these cytokines were also identified in heart tissue by measuring their mRNA levels. The RT-qPCR results showed that the mRNA expression levels of pro-inflammatory cytokines IL-6 and TNF-α were significantly reduced in heart tissue of mice with MI treated with *Ts*-AES (MI + AES) compared to the mice without treatment (MI + PBS) (7d: ANOVA, *F*_(3, 20)_ = 175.5, *P* < 0.0001; *F*_(3, 20)_ = 48.80, *P* < 0.0001; 14d: ANOVA, *F*_(3, 20)_ = 103.9, *P* < 0.0001; *F*_(3, 20)_ = 64.24, *P* < 0.0001; 28d: ANOVA, *F*_(3, 20)_ = 59.64, *P* < 0.0001; *F*_(3, 20)_ = 25.72, *P* < 0.0001, respectively) (Fig. 6a, b). Similarly, mRNA expression levels of IL-10 and TGF-β in heart tissue of MI mice treated with AES (MI + AES) demonstrated the same change pattern as in sera (Fig. 6c, d) with consistently increased IL-10 during the MI course (7d: ANOVA, *F*_(3, 20)_ = 88.39, *P* < 0.0001; 14d: ANOVA, *F*_(3, 20)_ = 97.92, *P* < 0.0001; 28d: ANOVA, *F*_(3, 20)_ = 51.56, *P* < 0.0001) and relatively increased TGF-β at the early stage of MI surgery (7d: ANOVA, *F*_(3, 20)_ = 20.39, *P* < 0.0001). The results suggest that treatment with *Ts*-AES systemically or in situ inhibits pro-inflammatory cytokine expression (TNF-α and IL-6) but stimulates the expression of regulatory cytokines (mainly IL-10) after MI has occurred, which is consistent with reduced pathological damage of infarcted heart tissue and improved heart tissue healing.

To determine whether macrophages and their phenotypes are involved in the inflammatory response or tissue repair in infarcted heart, the mRNA expression levels of the M1 macrophage marker iNOS and the M2 macrophage marker CD206 in the infarcted heart tissue were measured with RT-qPCR. The results showed that the mRNA expression levels of iNOS were highly stimulated in MI zone during the early stage of MI (Day 7 and Day 14) compared to mice with sham surgery but returned to normal at Day 28. There was no significant change in the mRNA expression of CD206 in MI heart tissue compared to that in sham surgery group. However, treatment with *Ts*-AES significantly reduced the mRNA expression of iNOS and increased the mRNA expression of CD206 in heart tissue of mice with MI (MI + AES) compared to MI mice without treatment (MI + PBS). Noticeably, the CD206 mRNA was also upregulated upon treatment of *Ts*-AES even in sham surgery group, indicating *Ts*-AES is a strong driver of M2 macrophages (7d: ANOVA, *F*_(3, 20)_ = 68.98, *P* < 0.0001; *F*_(3, 20)_ = 38.35, *P* < 0.0001; 14d: ANOVA, *F*_(3, 20)_ = 63.67, *P* < 0.0001; *F*_(3, 20)_ = 10.68, *P* = 0.0002; 28d: ANOVA, *F*_(3, 20)_ = 5.252, *P* = 0.0078; *F*_(3, 20)_ = 14.40, *P* < 0.0001, respectively) (Fig. 6e, f). The results strongly suggest that *Ts*-AES immunomodulate host immune system to drive macrophage polarization from M1 to M2 phenotype, which is correlated with the reduced pro-inflammatory cytokines and elevated regulator cytokines in heart tissues measured above, and consequently with the reduced heart pathology and boosted remodeling of heart structure and function after MI.

### *Ts*-AES induced M2 macrophage polarization in vitro

**Fig. 7:**
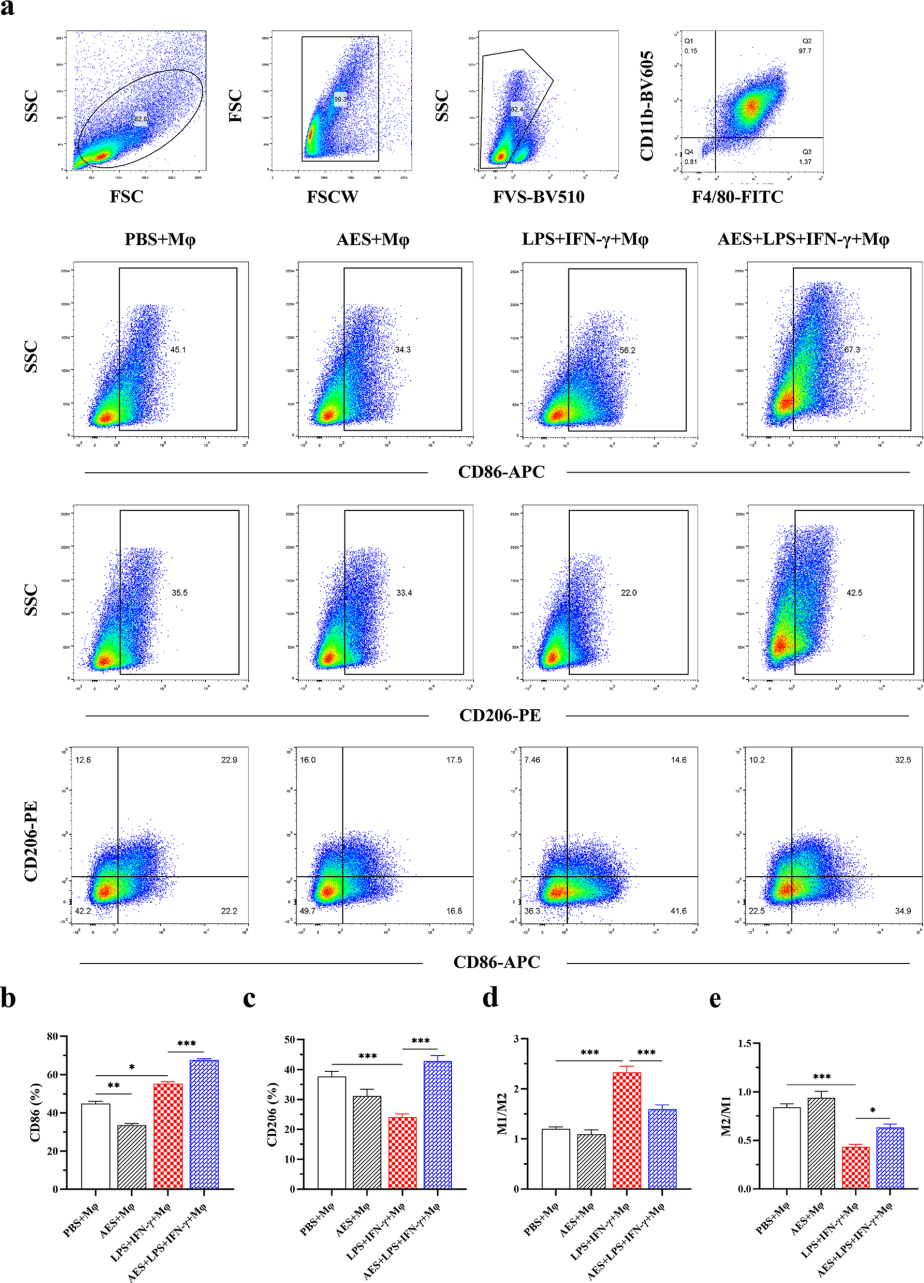
*Ts*-AES induced M2 macrophage polarization in vitro. **a** Flow cytometry was performed on mature BMDMs to sort CD11b^+^F4/80^+^ macrophages using FACS. BMDMs were incubated with *Ts*-AES (4 µg/ml), LPS (100 ng/ml) + IFN-γ (10 ng/ml), *Ts*-AES (4 µg/ml) + LPS (100 ng/ml) + IFN-γ (10 ng/ml) or PBS, respectively, for 24 h. The M1 (CD86) and M2 (CD206) markers were detected by FACS. **b** Percentage of CD86 (M1) in macrophages incubated with different stimulators. **c** Percentage of CD206 (M2) in different incubation groups. **d** M1/M2 ratio in different groups. **e** M2/M1 ratio in different groups (*n* = 6). Data are expressed as mean ± SEM, *P < 0.05, **P < 0.01, ***P < 0.001

To investigate the role of *Ts*-AES in macrophage polarization, the BMDMs were incubated with *Ts*-AES for 24 h and the changes of cell markers on BMDMs were detected by flow cytometry. The flow cytometry results revealed that *Ts*-AES significantly reduced F4/80^+^CD11b^+^CD86^+^ (M1) macrophages (AES + Mφ) compared to the group with PBS (PBS + Mφ). As a positive control, LPS + IFN-γ strongly stimulated F4/80^+^CD11b^+^CD86^+^ macrophages (M1) (ANOVA, *F*_(3, 20)_ = 215.6, *P* < 0.0001) (Fig. 7b). It was also noticed that *Ts*-AES itself had little effect on M2 macrophages (F4/80^+^CD11b^+^CD206^+^); however, *Ts*-AES strongly stimulated M2 macrophage polarization under inflammatory environment (under stimulation of LPS + IFN-γ) (ANOVA, *F*_(3, 20)_ = 20.32, *P* < 0.0001) (Fig. 7c), mimicking the inflammational environment induced by MI in this model.

### *Ts*-AES inhibited hypoxic cardiomyocyte apoptosis in vitro by inducing polarization of M2 macrophages

**Fig. 8:**
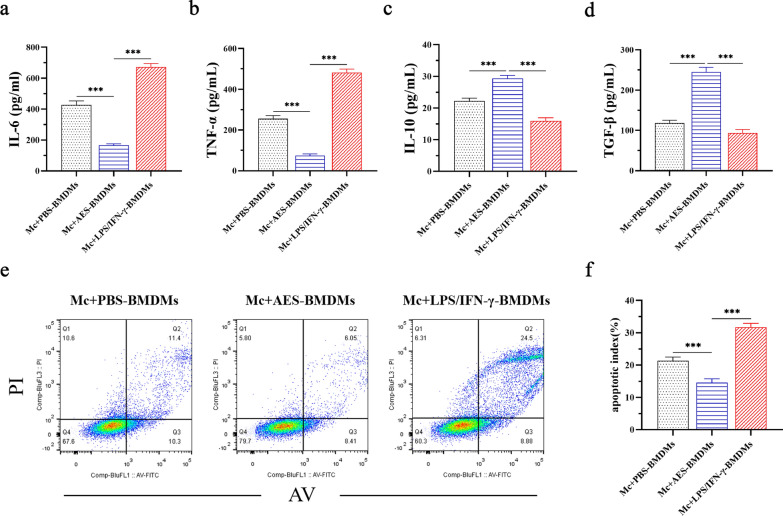
*Ts*-AES-treated BMDMs reduced hypoxia-induced cardiomyocyte apoptosis in vitro. Transwell technology was used to co-culture the primary cardiomyocytes with *Ts*-AES (4 µg/ml)-treated BMDMs, LPS (100 ng/ml) + IFN-γ (10 ng/ml)-treated BMDMs or non-treated BMDMs in a hypoxic environment for 24 h. ELISA was used to measure the levels of inflammatory cytokines (IL-6, TNF-α) and regulatory cytokines (IL-10 and TGF-β) in the culture supernatant (*n* = 6), and flow cytometry was used to detect the apoptosis rate of cardiomyocytes in each culture group (*n* = 4). Data are expressed as mean ± SEM, *P < 0.05, **P < 0.01, ***P < 0.001

To further determine the protective effect of *Ts*-AES-treated macrophages on hypoxic cardiomyocytes (Mc) in vitro, we examined the levels of inflammatory cytokines (IL-6, TNF-α) and regulatory cytokines (IL-10 and TGF-β) in the culture supernatants and the percentage of apoptotic cardiomyocytes after being co-cultured at hypoxia condition. After being incubated with *Ts*-AES-treated BMDMs (Mc + AES-BMDMs), the levels of IL-6 and TNF-α in the culture supernatant were significantly decreased (ANOVA, *F*_(2, 15)_ = 154.0, *P* < 0.0001; *F*_(2, 15)_ = 194.2, *P* < 0.0001, respectively) (Fig. 8a, b), while the levels of the regulatory cytokine IL-10 and TGF-β increased significantly (ANOVA, *F*_(2, 15)_ = 52.59, *P* < 0.0001; *F*_(2, 15)_ = 76.58, *P* < 0.0001, respectively) (Fig. 8c, d). Meanwhile, the percentage of apoptosis in hypoxic cardiomyocytes was significantly decreased when co-cultured with *Ts*-AES (Mc + AES-BMDMs) compared with culture without *Ts*-AES (Mc + PBS-BMDMs), while co-culture with LPS + IFN-γ significantly increased the apoptosis of hypoxic cardiomyocytes (Mc + LPS/IFN-γ-BMDMs) (ANOVA, *F*_(2, 9)_ = 53.61, *P* < 0.0001) (Fig. 8e, f). The results suggest that the *Ts*-AES-treated BMDMs reduce hypoxic cardiomyocyte apoptosis in vitro, possibly by stimulating M2 macrophages to reduce inflammatory cytokines and boost regulatory cytokines that protect cardiomyocytes from being damaged by anoxia.

## Discussion

The ischemia and hypoxia-caused inflammatory response is the main pathological mechanism of myocardial injury caused by MI. It determines the severity of MI and the subsequent ventricular remodeling. The immune cells mediated in the sterile inflammatory responses play dual roles in injury and protection during cardiac hypoxia and repair [46, 47]. It is critical to keep the balance of the pro- and anti-inflammatory pathways during the course of MI to reduce inflammation-caused heart tissue injury and promote the repair and remodeling of cardiovascular structure [17]. In this study, we attempted to use *Ts*-AES as an alternative treatment approach for MI based on their powerful immunomodulatory and regulatory functions identified before [48]. Treatment with *Ts*-AES significantly reduced mortality of MI, improved their cardiac functions and structure remodeling and alleviated inflammatory response and organ damage in infarcted heart tissue. The promising results provide a novel approach to use helminth-derived immunomodulatory proteins in the treatment of inflammatory diseases or tissue injury such as MI.

Atherosclerosis causing thromboembolism is considered the most common cause of MI [49]. To create a mouse model of MI, we ligated LAD to block myocardial blood flow, successfully mimicking the pathology of MI in clinical sector. In this study, we establish a MI mouse model and observed MI-induced cardiac injury in mice, mainly in the form of structural and functional damage accompanied by elevated levels of proinflammatory factors in blood and tissues, which is very similar to the pathophysiological features of MI observed in clinical patients.

As previously described, macrophages undergo a transition from early pro-inflammatory to late anti-inflammatory effects after MI [16]. However, prolonged pro-inflammatory response after MI can lead to serious pathological consequences, including cardiomyocyte death, compromised systolic function, atrial dilatation due to extensive matrix degradation, compromised ventricular wall integrity, cardiac rupture and fibrosis [11, 50]. M1 macrophage-secreted TNF-α causes degradation of collagen and induces extracellular matrix in the MI region, leading to thinning of the ventricular wall and increasing the likelihood of cardiac rupture [51]; IL-6 can lead to inflammatory factor storm [52, 53]. A large release of TNF-α and IL-6 in MI area worsens the myocardial damage during acute MI, decreases myocardial viability and leads to unfavorable ventricular remodeling (VR) pairs. In this study, we found elevated levels of M1 macrophage-associated pro-inflammatory cytokines (TNF-α and IL-6) and their mRNA expression in cardiac tissues on days 7, 14 and 28 after MI surgery, along with elevated mRNA levels of the M1 macrophage marker iNOS in cardiac tissues (Figs. 5, 6). This M1 macrophage-induced inflammation in ischemic heart tissue is consistent with decreased survival, impaired cardiac function and inflammatory cell infiltration and pathological damage in cardiac tissues in mice. Therefore, early regulation of inflammatory factor secretion to create a microenvironment conducive to ventricular repair is extremely important to alleviate MI and its subsequent excessive ventricular remodeling.

Recent studies have shown that helminth infection and helminth-derived proteins can immunomodulate the host immune system to alleviate different inflammatory diseases such as arthritis [54], colitis [55–57], polymicrobial sepsis and sepsis caused key organ damage [31, 58], especially heart damage [37]. In this study, we found that treatment with *Ts*-AES in mice with MI induced the polarization of mouse macrophages from M1 to M2 type, associated with significantly reduced inflammation in MI tissue and improved heart tissue repair and remodeling. Particularly *Ts*-AES significantly inhibited LPS + IFN-γ-induced M1 polarization of BMDMs and promoted M2 macrophage polarization as evidenced by a decrease in the percentage of F4/80^+^CD11b^+^CD86^+^ type macrophages and an increase in the percentage of F4/80^+^CD11b^+^CD206^+^ type macrophages after *Ts*-AES stimulation (Fig. 7). We also found that *Ts*-AES-induced polarization of M2 macrophages in vitro could inhibit apoptosis of hypoxic cardiomyocytes (Fig. 8). It supports the therapeutic effect of *Ts*-AES in mice with MI in vivo. Similar effects of *Ts*-AES on macrophage polarization from M1 to M2 phenotype were also found in vivo in MI mice, characterized by decreased levels of M1-related markers iNOS and pro-inflammatory cytokines TNF-α and IL-6 mRNA expression and increased levels of M2-related markers CD206 and regulatory cytokines IL-10 mRNA expression in the heart tissue of treated mice (Fig. 6), even though there was no significant change of TGF-β expression level in *Ts*-AES treated mice with MI observed in this study. These results correlated with the reduction of pathological damage in mouse cardiac tissue with MI, improvement of cardiac function and reduction of mortality when treated with *Ts*-AES, suggesting that the therapeutic effect of *Ts*-AES on MI is mostly related to *Ts*-AES-promoted macrophage polarization from M1 phenotype to M2 phenotype in the damaged tissue.

After MI, many macrophages accumulate at the site of myocardial injury, among which M2 macrophages activate cardiac fibroblasts into collagen-secreting myofibroblasts by releasing matrix metalloproteinase (MMP) [59] and TGF-β (serves as the master switch) regulating the transition from inflammation to fibrosis, which are involved in scar formation and cardiac fibrosis [7, 60, 61]. Meanwhile, myofibroblasts release cytokines TGF-β, angiotensin II (Ang-II) and platelet-derived growth factor (PDGF) in an autocrine manner, which further stimulate cardiac fibroblasts to transform into myofibroblasts that express α-SMA in large amounts to further promote myocardial fibrosis to prevent cardiac rupture [62–64]. However, excessive activation of cardiac fibroblasts can lead to scar proliferation, reduce cardiac systolic and diastolic function, and trigger heart failure [14]. Therefore, it is important to induce M2 macrophages that exert antifibrotic activity in late MI to inhibit excessive activation of cardiac fibroblasts, reduce scar tissue formation and avoid adverse VR [65]. In this study, we found that there was no significant change in α-SMA mRNA expression in the MI area of mice treated with *Ts*-AES at day 7 after MI, whereas the mRNA expression of α-SMA was significantly decreased on days 14 and 28 after MI, which is consistent with the reduced cardiac fibrosis observed in MI mice treated with *Ts*-AES (Fig. 4). The reduced fibrosis in MI area was also supported by the reduced TGF-β expression levels in serum and heart tissue after being treated with *Ts*-AES (Figs. 5, 6). At the same time, M2 macrophages release vascular endothelial growth factor (VEGF) [66], which promotes neovascularization, alleviates tissue ischemia and hypoxia, reduces myocardial injury and accelerates ventricular repair [67, 68]. In this study, the expression level of VEGF in the myocardial infarct area was significantly increased in MI mice treated with *Ts*-AES, indicating that *Ts*-AES promotes the neovascularization in the myocardial infarct area of mice with MI. IL-10 secreted by M2 macrophages is a potent anti-inflammatory cytokine with the ability to suppress synthesis of proinflammatory cytokines and chemokines in macrophages through activation of STAT3 signaling [69] and improves cardiac remodeling after myocardial infarction by stimulating M2 macrophage polarization and fibroblast activation [70, 71]. In the present study, the levels of IL-10 were significantly increased in *Ts*-AES-treated MI mice and reached to the highest level on day 14 after MI, indicating that IL-10 is mainly involved in the regulation in the middle stage of MI.

In our previous study, we demonstrated that both ES products from *T. spiralis* adult worms (AES) or muscle larvae (MES) provided therapeutic effects on sepsis-induced heart injury [37] or acute lung injury [31], indicating that some components in adult or larva ES products play immunomodulatory roles in host immune response to reduce inflammatory responses. Both AES and MES reduced pro-inflammatory cytokines (TNF-α, IL-6) and induced regulatory cytokines (IL-10 and TGF-β). The role of MES may act by inhibiting HMGB1/TLR2/MyD88 inflammatory signal pathway [37]. In this study, we have demonstrated for the first time the therapeutic effect of *Ts*-AES on MI through modulation of macrophage polarization from M1 to M2. The different effects of AES and MES indicate that *T. spiralis* ES products contain a variety of proteins that may have different biological and immunological functions. It is important to identify those immunomodulatory proteins in AES and MES and develop them as therapeutic agents for the treatment of inflammatory diseases such as MI-induced inflammation. The worm-derived raw materials are not suitable as pharmaceutical reagents because of their safety issue and the difficulties of scale-up production of the products [48, 72]. Therefore, it is necessary to identify specific molecules in the *Ts*-ES complex that are involved in immunomodulation. Current studies have identified various protein components in *Ts*-AES with potential regulatory functions, e.g. serine protease from adult stage of *T. spiralis* alleviates the severity of TNBS-induced colitis by balancing CD4^+^ T cell immune response [73]; recombinant *T. spiralis* cystatin alleviates polymicrobial sepsis through activating regulatory macrophages [58]. Also, the inflammatory signaling pathway by which *Ts*-AES regulates macrophage polarization to improve MI is still not clear. Our next step is to identify specific and effective components in the *Ts*-AES that regulate macrophage polarization and exert a therapeutic effect in MI or other inflammatory diseases.

## Conclusion

*Ts*-AES improved cardiac function and ventricular remodeling in mice with myocardial infarction by inducing regulatory macrophage (M2) polarization. The results of this study suggest that worm-derived proteins may be used as pharmaceutical agents to treat myocardial injury or other inflammatory diseases.

## Data Availability

All datasets presented in this study are included in the article/supplementary material.

## Abbreviations

MI: Myocardial infarction
VR: Ventricular remodeling
SV: Stroke volume
LVEF: Left ventricular ejection fraction
LVFS: Left ventricular fractional shortening
LVESV: Left ventricular end systolic volume
LVEDV: Left ventricular end diastolic volume
LV MPI: Left ventricular myocardial performance index
ELISA: Enzymelinked immunosorbent assay
IL-6: Interleukin 6
TNF-α: Tumor necrosis factor-alpha
TGF-β1: Transforming growth factor-β1
IL-10: Interleukin 10
α-SMA: Alpha-smooth muscle actin
VEGF: Vascular endothelial growth factor
iNOS: Inducible nitric oxide synthase
